# Tomoscopy: Time‐Resolved Tomography for Dynamic Processes in Materials

**DOI:** 10.1002/adma.202104659

**Published:** 2021-09-23

**Authors:** Francisco García‐Moreno, Paul Hans Kamm, Tillmann Robert Neu, Felix Bülk, Mike Andreas Noack, Mareike Wegener, Nadine von der Eltz, Christian Matthias Schlepütz, Marco Stampanoni, John Banhart

**Affiliations:** ^1^ Institute of Applied Materials Helmholtz‐Zentrum Berlin für Materialien und Energie Hahn‐Meitner‐Platz 1 Berlin 14109 Germany; ^2^ Institute of Materials Science and Technology Technische Universität Berlin Hardenbergstr. 36 Berlin 10623 Germany; ^3^ Swiss Light Source Paul Scherrer Institute Forschungsstr. 111 Villigen 5232 Switzerland; ^4^ Institute for Biomedical Engineering ETH Zürich Gloriastrasse 35 Zürich 8092 Switzerland

**Keywords:** combustion, metal foams, solidification, time‐resolved, tomography, tomoscopy, X‐rays

## Abstract

The structure and constitution of opaque materials can be studied with X‐ray imaging methods such as 3D tomography. To observe the dynamic evolution of their structure and the distribution of constituents, for example, during processing, heating, mechanical loading, etc., 3D imaging has to be fast enough. In this paper, the recent developments of time‐resolved X‐ray tomography that have led to what one now calls “tomoscopy” are briefly reviewed A novel setup is presented and applied that pushes temporal resolution down to just 1 ms, that is, 1000 tomograms per second (tps) are acquired, while maintaining spatial resolutions of micrometers and running experiments for minutes without interruption. Applications recorded at different acquisition rates ranging from 50 to 1000 tps are presented. The authors observe and quantify the immiscible hypermonotectic reaction of AlBi10 (in wt%) alloy and dendrite evolution in AlGe10 (in wt%) casting alloy during fast solidification. The combustion process and the evolution of the constituents are analyzed in a burning sparkler. Finally, the authors follow the structure and density of two metal foams over a long period of time and derive details of bubble formation and bubble ageing including quantitative analyses of bubble parameters with millisecond temporal resolution.

## Graphical abstract

Recent developments of time‐resolved X‐ray tomography, called “tomoscopy,” are presented. Tomoscopy provides up to 1000 tomograms per second at µm‐range resolution, running experiments for minutes without interruption. Tomoscopy is applied to study various dynamic processes in materials science.

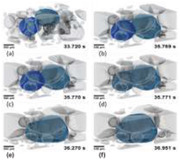

## 1 Introduction

X‐ray tomography is often applied to investigate the structure of matter non‐destructively since it provides the precise spatial arrangement of internal constituents. Medicine is probably the most well‐known discipline that has taken advantage of this method and has pushed forward its development.^[^ ^1^, ^2^, ^3^ ^]^ However, tomography has also found application in other research areas such as materials science,^[^ ^4^, ^5^ ^]^ biology,^[^ ^6^ ^]^ archaeology,^[^ ^7^ ^]^ or even fluid dynamics^[^ ^8^ ^]^ and is also gaining more and more acceptance in industry, for example, for quality control^[^ ^9^ ^]^ or non‐destructive testing.^[^ ^10^ ^]^ The combination of image acquisition with real‐time reconstruction algorithms,^[^ ^11^ ^]^ advanced image analysis,^[^ ^12^ ^]^ feature segmentation, and recognition analysis algorithms^[^ ^13^, ^14^ ^]^ with modern machine learning tools^[^ ^15^, ^16^ ^]^ is enhancing the potential of this method. Laboratory scanners are nowadays widespread and powerful and benefit from improved spatial and temporal resolutions, although cutting‐edge experiments are still restricted to highly brilliant synchrotrons and X‐ray free‐electron lasers. Very short acquisition times at high spatial resolutions are available.^[^ ^17^, ^18^ ^]^ The desire for high spatial and temporal resolutions, large fields of view and high total recording time implies a conflict of goals. An overview of actual speeds and resolutions available at different facilities has been given in the literature.^[^ ^19^, ^20^, ^21^ ^]^

For the analysis of dynamic processes, temporal resolution is the most important parameter to optimize, but without compromising too much of others such as spatial resolution, field of view, and total possible acquisition period. Time‐resolved tomography has a long and unsystematic history of nomenclature. A large number of publications characterized by attributes such as high‐speed, rapid‐acquisition, fast, very fast, ultrafast, real‐time, time‐resolved, live, 4D, cinetomography, high‐throughput, millisecond‐order, sub‐s, time‐lapse, and temporal, can be found in the literature, and others such as in situ, in vivo, and operando, which indirectly imply a certain temporal resolution. Recently, we proposed the term tomoscopy for time‐resolved tomography in analogy to radioscopy and quantify the number of tomograms per second (tps) in analogy to the term frames per second (fps) used to describe 2D image sequences.^[^ ^22^ ^]^ In fact, the term tomoscopy has already been employed in 1970 for the observation under different angles of rapidly repeated X‐ray exposures.^[^ ^23^ ^]^ Later, in 1983, the corresponding acquisition velocity was improved up to 50 tps with 16 projections per tomogram by avoiding the use of mechanical movements, which is of special relevance in medicine and flow dynamics.^[^ ^24^ ^]^ There, a superposition of images on a television screen was obtained as fast digital recording or computer analyses were unavailable. In any case, the purpose of tomoscopy was, and is, to gain time‐resolved 3D information of a structure with sufficient spatial resolution, field of view, and recording time.^[^ ^22^ ^]^

Evolving metallic foams have been studied by the current authors with in situ X‐ray radioscopy at acquisition rates up to 105 000 fps to analyses liquid film rupture and bubble coalescence.^[^ ^25^, ^26^, ^27^, ^28^ ^]^ X‐ray radioscopy was also widely applied in studies of the solidification behavior of metallic alloys.^[^ ^29^, ^30^ ^]^ Now that tomoscopy allows for tomographic acquisition rates similar to the ones of previous radioscopic studies,^[^ ^22^ ^]^ the analyses are taken to the next level. Its main advantage compared to radioscopy lies especially in the acquisition of time‐resolved full 3D information instead of the convoluted information in radiographic projections, in the possibility of a wide range of quantitative analyses, and in the study of bulk samples while avoiding edge effects of flat samples that occur in almost any 2D radioscopy.

Recent developments in time‐resolved imaging at different synchrotron radiation sources are focused on a combined spatio‐temporal resolution for dynamic analyses.^[^ ^19^ ^]^ For X‐ray radioscopy, very short exposure times (down to 100 ps per image, 1 million fps) are now possible by taking advantage of high‐intensity single‐bunch radiation,^[^ ^18^, ^31^, ^32^ ^]^ but the total acquisition period is very restricted due to the available camera recording technologies. Furthermore, X‐ray free‐electron lasers allow for an image acquisition in the femtosecond range, but for single images only.^[^ ^33^ ^]^ X‐ray tomoscopy studies have allowed for sub‐second acquisition times since 2011.^[^ ^19^, ^34^, ^35^, ^36^, ^37^, ^38^, ^39^, ^40^ ^]^ This method has been applied by our group in the past years, especially to evolving metallic foams.^[^ ^22^, ^40^, ^41^, ^42^, ^43^ ^]^ In situ analyses of metallic alloy solidification are also of special interest.^[^ ^44^, ^45^, ^46^, ^47^ ^]^

In this work, we present a tomoscopy setup with spatial resolutions in the µm‐range, a field of view of several square millimeters, maximum continuous acquisition periods in the range of minutes and acquisition rates up to 1000 tomograms per second, and all this simultaneously. We demonstrate the potential of tomoscopy by reporting the results of several case studies in the order of increasing acquisition rate. These include two solidifying alloys (AlBi10 and AlGe10), the highly exothermic combustion reaction of a burning sparkler, the gas nucleation, bubble growth, and structure evolution of a thixocast AlSi6Cu4 foam, and the coalescence of two bubbles in an evolving AlSi8Mg4 foam. The measurements and associated results have not been reported before.

## 2 Experimental Section

### 2.1 Tomoscopy Environment

#### 2.1.1 Tomoscopy Setup

**Figure 1:**
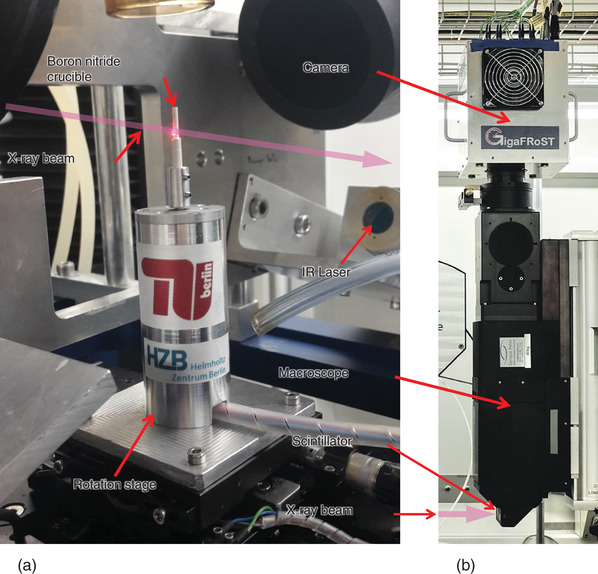
Tomoscopy setup installed at the TOMCAT instrument of the SLS, PSI comprising: a) A self‐developed high‐speed rotation stage (operational up to 500 Hz, i.e., allowing for up to 1000 tps) and a hollow cylindrical boron nitride crucible into which the samples are inserted. Heating of the samples is provided by two 150 W IR lasers placed in perpendicular to the X‐ray beam illustrated by a pink arrow here. b) Position of the LuAG:Ce scintillator screen, the optical macroscope, and the GigaFRoST CMOS camera on top.

Tomoscopy experiments were performed at the TOmographic Microscopy and Coherent rAdiology experimenTs (TOMCAT) beamline X02DA of the Swiss Light Source, Paul Scherrer Institute, Switzerland. 5 mm of glassy carbon and a 325‐µm thick single crystalline Si wafer were used to filter the polychromatic beam produced by a 2.9 T bending magnet and to suppress lower energies and reduce the heat load. The resulting beam was therefore a filtered white beam, which is described in more detail in the literature.^[^ ^48^ ^]^ The transmitted intensities were converted to visible images by a 150‐µm thick LuAG:Ce scintillator (Crytur, Czech Republic) at a variable distance of 150–260 mm from the sample, allowing for simultaneous absorption and phase contrast. The images were magnified by a high‐numerical‐aperture macroscope (Optique Peter, Lentilly, France) with a magnification fixed at 4×^[^ ^49^ ^]^ and recorded by the “gigabit fast readout system for tomography” (GigaFRoST) high‐speed CMOS camera (see **Figure** **1**).^[^ ^50^ ^]^ The resulting effective pixel size was 2.75 µm. This resulted in a measured spatial resolution of 7.6 µm at 100 tps and 8.2 µm at 1000 tps (Figure S1, Supporting Information). Some relevant acquisition parameters for the different case studies are listed in **Table** **1**.

**Table 1:** Acquisition parameters for the different case studies. Columns represent: acquisition rate of tomograms per second, no. of projections in each tomogram, exposure time for each radiogram, acquisition rate of radiograms in kilo‐frames per second (kfps), field of view, number of tomograms in entire experiment, data set size of the raw projection series, size of total data set after reconstruction of all time steps, and radial acceleration in *g*‐units at the outmost point of sample

Experiment^a)^	Tps [s^−1^]	No. projections	Exposure [ms]	kfps	FoV [pixel]	No. of tomos	Raw file size [GB]	Rec. file size [GB]	Acceleration [*g*]
AlBi10	50	200	0.09	10	1008 × 400	3600	541	2725	2.5
AlGe10	200	100	0.045	20	528 × 280	11 801	331	1716	20.1
Sparkler	400	100	0.02	40	528 × 128	9501	121	1381	129
AlSi6Cu4 foam	650	62	0.02	40	480 × 128	44 354	315	2436	212
AlSi8Mg4 foam	1000	40	0.02	40	528 × 120	43 899	208	2735	503

^a)^ All experiments done with filtered white beam, 5 mm of glassy carbon, 325 µm‐thick Si filter, and 2.75 µm pixel size.

A self‐developed high‐speed rotation stage with a very low eccentricity and a high angular speed stability now allowed for an operation at up to 500 Hz rotation speed, corresponding to 1000 tps (40 projections, 1 ms for each full tomogram corresponding to 180° rotation). The stage was based on a brushless servo‐motor with a corresponding speed and positioning controller, both from Faulhaber, Germany, the rotation of which could be synchronized with the acquisition velocity of the recording camera.

Two consecutive tomograms always represented a different angle range, that is, 0–180° for the first and 180–360° for the following. This gave rise to the slight jitter observed in some of the videos in the supplement, which, however, did not have a negative impact on the quantitative analyses presented in this paper.

#### 2.1.2 Spatio‐Temporal Resolution

**Figure 2:**
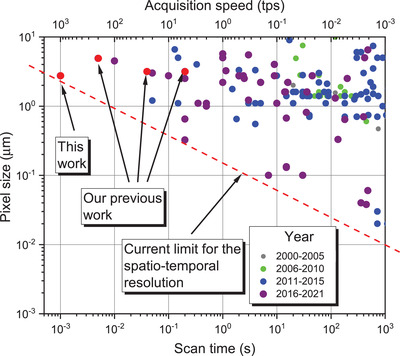
Temporal versus spatial resolution expressed by pixel size versus scan time or acquisition speed of time‐resolved tomography experiments in materials science since the year 2000. Red broken line denotes the current limit of spatio‐temporal resolution. All data was collected from the databases Fast‐Tomography^[^ ^20^ ^]^ and Tomoscopy Resolution^[^ ^51^ ^]^ and adapted from ref. [19]. Some current and previous work of the authors is marked by red dots.^[^ ^22^, ^40^, ^42^ ^]^

In **Figure** **2**, the state‐of‐the‐art experimental parameters concerning spatial and temporal resolution in materials science extracted from the literature are summarized. Current improvements of X‐ray flux and sensitivity of the acquisition equipment had allowed for a pronounced improvement in temporal resolution down into the millisecond range while keeping spatial resolution in the µm range. The merit of the system presented in this work was mainly achieved by the combination of highly brilliant synchrotron light, a dedicated beamline, a highly performant optical macroscope, a high‐end data recording system, and a new fast and precise rotation stage. The inverse correlation between spatial and temporal resolution was responsible for the actual acquisition limits, achieved mainly in the past 5 years and shown by a red broken line in Figure 2.

#### 2.1.3 Laser Heating

Samples were heated up using two 150 W infrared lasers situated almost in the opposite direction and perpendicular to the X‐ray beam (see Figure 1).^[^ ^46^ ^]^ The heating and cooling temperature profiles were adjusted indirectly through the laser power and measured on the outer surface of a boron nitride crucible by a calibrated pyrometer. The system is described in more detail elsewhere.^[^ ^22^, ^46^ ^]^

#### 2.1.4 Data Handling

The demands on the data acquisition system required for tomoscopy were very high. Typical recording speeds of 10–40 kHz frame rate had to be sustained in some cases over minutes, depending on the experiment. The dedicated GigaFRoST camera and readout system had been developed precisely to overcome this problem.^[^ ^50^ ^]^

All the projections were recorded continuously with up to ≈8 GB s^−1^ transfer rate. Blocks belonging to an angular range of 180° were extracted, filtered using the propagation‐based phase contrast algorithm of Paganin et al.^[^ ^52^ ^]^ with a δ/β ratio adapted to the respective material system and then reconstructed with the “gridrec” algorithm.^[^ ^11^ ^]^ The number of projections was adjusted via the frame rate of the high‐speed camera to the respective rotation speeds and ranged between 40 and 200 per tomogram. This number was sufficient for reconstruction and segmentation as the sample radius was limited in width to 0.5–1 mm.

To determine the dendritic growth rate of a solidifying alloy, the reconstructed intensity values of a line located in the center of a given dendrite along its growth direction were obtained and the spatial positions of the grey value transition from solid to liquid phase identified. To obtain the relative material density of a foam, the intensities of the reconstructions normalized to the grey values of the liquid and bubbles were averaged over the sample section height and displayed as a function of their radial distance from the rotation axis.

Further data processing depended on the respective experiment and usually started with a spatio‐temporal filtering and segmentation of the regions of interest in the reconstructed volume. Segmented objects were characterized by their position, size, shape, and orientation, and the rate of change could be measured. There were some limits to the methods of evaluation that depended on the respective problem and could not be described in general terms.

### 2.2 Sample Preparation

#### 2.2.1 Casting Alloys

Two aluminum‐based casting alloys were prepared. The first was AlGe10 (all compositions given in wt%) and the second AlBi10, for which 10.8 g aluminum was alloyed with 1.2 g bismuth or germanium, respectively, with all ingredients being 5N pure. For the AlGe10 alloy, aluminum was fused in an induction furnace by continuously increasing the heating power, after which the alloying element was added to the levitating, molten aluminum and the power readjusted until a spherical melt was created. The melt was stirred in a magnetic field for about 30 min to create a homogeneous solution. After solidification, the alloy was melted a second time and further homogenized for 30 min followed by subsequent fast solidification in a water‐cooled copper crucible. AlBi10 melt was prepared in a graphite crucible by first melting aluminum at 750 °C for 30 min and then adding bismuth at 800 °C and holding for 30 min. To assure adequate mixing, the melt was constantly stirred with a graphite rod. The melt was then cast into a steel mold of 12 mm diameter and 150 mm length. From the resulting AlGe10 and AlBi10 alloy beads of 1 and 2 mm diameter, respectively, and 5 mm length, samples were prepared from their interior (to avoid surface effects and concentration gradients) by CNC milling or electric discharge machining to fit into the boron nitride crucible that was mounted on the tomography rotation stage.

#### 2.2.2 Sparkler

A commercial sparkler of ≈3 mm diameter made of a mixture of iron, aluminum, barium nitrate, potassium chlorate, and cellulose around a steel wire was shortened to ≈20 mm and mounted on a steel holder that was fixed to the rotation stage. Imaging was performed on the top of the sparkler in a region without a steel wire inside, which would disturb imaging due to its high X‐ray absorption. The top part of the sparkler with a diameter of 1.6 mm was selected in order to make the sample fit inside the available field of view. The sparkler was ignited remotely using the IR lasers mentioned above.

#### 2.2.3 Metal Foams

Elemental aluminum, silicon, and pre‐alloyed AlMg50 powders together with 0.25 wt% TiH_2_ powder acting as a foam blowing agent were mixed for 20 min. To obtain an AlSi8Mg4 foamable precursor the powder blend was then cold‐compacted in a steel die of 36 mm diameter at 300 MPa for 5 s, followed by a hot‐compaction step at 400 °C for 15 min. This procedure represented a standard powder metallurgical compaction routine described elsewhere in more detail.^[^ ^53^ ^]^ Another precursor was prepared from elemental powders to yield AlSi6Cu4 alloy by semi‐solid processing as described elsewhere.^[^ ^54^ ^]^ Such precursors were known to evolve into a more uniform foam, however, of lower stability than foams made from powder compacts. The authors utilized such less stable foams for test purposes in this contribution. From both types of dense foamable precursors, cylindrical samples of 2 mm diameter and 5 mm length were cut to fit in the boron nitride crucible (Figure 1). The crucible was mounted on the high‐speed rotation stage and foaming was achieved by heating up the crucible with the two infrared lasers.

## 3 Results and Discussions

### 3.1 Solidification of AlBi10 Resolved at 50 tps

Monotectic alloys with a stable miscibility gap in the liquid state such as Al–Bi*X* with *X* = 3–10 wt% of bismuth are commercially available materials.^[^ ^55^ ^]^ Hypermonotectic compositions (*X* > 3.4 wt%) are used as master alloys and hardeners for improving machinability^[^ ^56^ ^]^ and also find potential application as bearing materials in engines (cars, ships),^[^ ^57^, ^58^ ^]^ where soft particles (here bismuth) are embedded in a harder aluminum matrix. Monotectic alloys are in general difficult to cast and, especially in this particular case of a large density ratio of more than 3.6 between bismuth and aluminum, liquid Bi‐rich droplets precipitate in the aluminum melt and segregate due to gravity.

**Figure 3:**
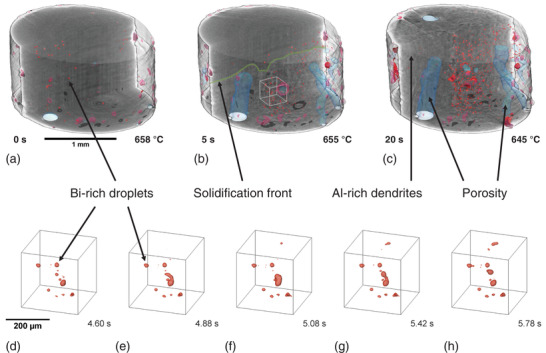
a–c) 3D rendered images of a cylindrical AlBi10 sample in different stages of solidification measured with tomoscopy at 50 tps. The matrix is set to transparent in the front half of the cylindrical sample. a) Bi‐rich droplets (in red) coexist with the melt. b) The solidification front (shown as a green line) advances through the whole sample from the bottom to the top in about 20 s at a mean velocity of 130 µm s^−1^. Bi‐rich droplets get trapped between the growing Al‐rich dendrites and porosity (in blue) forms. c) The number and volume of the Bi‐rich droplets increase during solidification and further porosity (in blue) forms. d–h) Magnified extracted volumes as marked by a white cube in (b) shows Bi‐rich droplets interacting in (d), merging first in (e) to (f), but separating later in (g) and (h) in a short time interval of about 1 s at around 655 °C (Video S1, Supporting Information).

Tomoscopy of a cylindrical AlBi10 sample (2 mm in diameter) during solidification at a cooling rate of 0.7 K s^−1^ was performed in situ with 50 tps (**Figure** **3**). The apparition of the solidification front in the field of view advancing from the bottom to the top defines *t*_0_ = 0 s (Figure 3a). The hypermonotectic AlBi10 composition forms a homogenous liquid phase, *L*, completely miscible above its binodal temperature *T*_bin_ ≈ 800 °C according to the binary phase diagram (see Figure S3, Supporting Information).^[^ ^59^ ^]^ Below *T*_bin_ the liquid phase undergoes a spinodal decomposition into two immiscible liquids (*L* → *L*_1_ + *L*_2_). Bi‐rich *L*_2_ droplets nucleate and grow by diffusion in the Al‐rich *L*_1_ matrix melt, and move and interact with each other in the melt. This is clearly seen in both Figure 3 and Video S1, Supporting Information. The progressing precipitation of Bi‐rich droplets shown in red in Figure 3 takes place prior to the monotectic reaction (*L*_1_ → α‐Al + *L*_2_ at the monotectic temperature *T*_mon_ = 657 °C). For another experiment performed at a cooling rate of 4 K s^−1^, first droplets were found at 682 °C, corresponding to an undercooling of 118 K. This value is much higher than the 20 K undercooling estimated by Wu et al.,^[^ ^60^ ^]^ but in accordance with the Δ*T* = 70 and 110 K found by Schaeffer et al. for AlBi6 and AlBi8, respectively.^[^ ^61^ ^]^

The droplets are driven by hydrodynamic forces, gravity, melt convection, and Marangoni forces as described in the literature and also observed in X‐ray radioscopy experiments.^[^ ^60^, ^61^, ^62^ ^]^ Furthermore, *g*‐forces caused by the rotation of the sample with 25 Hz in the course of tomoscopy image acquisition, which should act more on the denser Bi‐rich droplets depending on their radial position, could reach values of up to 2.5 × *g* at the edge of the container, radius 1 mm (Figure S2, Supporting Information), and influence their motion in the liquid melt even more than natural gravity.^[^ ^61^ ^]^

Some large Bi‐rich droplets segregate to the sample surface irrespective of rotation as shown in Figure 3 and by Lu et al.^[^ ^63^ ^]^ From the image of the sample at 645 °C (Figure 3c), we see that most of the bismuth precipitations are located near the sample center after being trapped between the growing dendrites, suggesting that other parameters play a more important role, for example, the radial temperature distribution during solidification. Therefore, the influence of centrifugal forces can be neglected in this particular case.

Upon further cooling to below *T*_mon_, Al‐rich dendrites form and extend into the melt from the bottom to the top as the monotectic conversion front advances. We observe the progression of the solidification front and denote it by a green line on the cross section in the middle of the tomogram in Figure 3b. Its mean velocity is 130 µm s^−1^ at a cooling rate of 0.7 K s^−1^, corresponding to a motion of 2.6 µm (≈1 voxel size) per tomogram. This demonstrates that the applied acquisition rate of 50 tps is necessary: Slower acquisition would lead to massive blurring of the small particles. During dendrite growth simultaneously with the monotectic reaction we observe further precipitation of Bi‐rich droplets, which get trapped between the Al‐rich dendrites. The final microstructure is composed of Al‐rich dendrites, porosity, and bismuth precipitates trapped in between (Figure 3c). During and after solidification of the matrix, bismuth continues to diffuse to the already precipitated droplets, increasing their volume and agglomerating as can be observed by comparing Figure 3b,c. This ageing process resembles an Ostwald ripening process (see Figure S4, Supporting Information, and discussion there). A magnified section marked in Figure 3b by a white cube is presented in Figure 3d–h and shows in detail the merger and separation of two Bi‐rich droplets constrained between solid Al‐rich dendrites in a time interval of ≈1.2 s. The droplet–droplet interaction found here (Figure 3d–h and Video S1, Supporting Information) can be explained by hydrodynamically driven coalescence and repulsion as reported in the literature, where at least seven mechanisms have been proposed.^[^ ^64^, ^65^, ^66^ ^]^ A possible explanation of the coalescence and unexpected separation of two Bi‐rich droplets could be found by the repulsive diffusion‐coupling mechanism as proposed by Schaffer et al.^[^ ^66^ ^]^

The voids formed during solidification due to precipitation of gas advance in parallel to the solidification front and evolve into elongated pores. The corresponding gas–solid interfaces are shown in light blue in Figure 3a–c. Gas bubbles, probably filled with hydrogen, prevent nucleation of Bi in their vicinity as was shown by Lu et al.^[^ ^63^ ^]^ A small amount of Bi‐rich liquid phase remains and finally, at the melting temperature of Bi (*T*_Bi_ = 270 °C), further Bi‐rich droplets precipitate and eventually solidify according to the reaction *L*_2_ → α‐Al + Bi.^[^ ^61^ ^]^

### 3.2 Solidification of AlGe10 Resolved at 200 tps

The microstructure of alloys largely determines their properties. To improve the performance of an alloy it is mandatory to reveal the mechanisms governing microstructure formation and evolution during solidification. To overcome the opacity of metals, in situ X‐ray radioscopy and tomography studies have been successfully performed in the past.^[^ ^29^, ^30^, ^67^ ^]^ Al–Ge behaves similarly to the commercially relevant Al–Si casting alloy system.^[^ ^68^ ^]^ Al–Ge, together with Al–Cu alloy, belongs to the most common systems studied as it provides very good X‐ray contrast between the phases. The ratio of the elemental attenuation coefficients is around 20 for Al–Ge and 30 for Al–Cu for photon energies right above the absorption K‐edge of the respective heavier component, namely ≈11 and ≈9 keV, respectively.^[^ ^69^ ^]^ Research has mostly focused on columnar and equiaxed solidification as well as on the corresponding transition between both solidification modes.^[^ ^70^, ^71^, ^72^, ^73^, ^74^ ^]^ However, 2D radioscopy studies require samples of 100–200 µm thickness in the direction of the X‐ray beam to avoid too much superimposition of image features. This leads to pronounced effects of the container walls such as i) heterogeneous nucleation at crevices in or oxide skins resting at the crucible walls^[^ ^75^, ^76^ ^]^ or ii) constrained dendrite growth. Such studies could therefore yield different results as one would expect for more bulky samples.

We used tomoscopy to study the columnar solidification process and microstructure evolution of a cylindrical AlGe10 sample of 1 mm diameter with sufficient temporal resolution. The applied cooling rate of 17 K s^−1^ corresponds to realistic casting conditions^[^ ^77^, ^78^ ^]^ unlike previous radioscopic studies where the cooling rates were much lower, for example, ≈1 K min^−1^.^[^ ^79^, ^80^, ^81^ ^]^ Recently published work, for example from Feng et al., who studied a solidifying Fe‐rich intermetallic compound with radioscopy at 4 K s^−1^, is directed toward higher rates, but 3D information is still lacking.^[^ ^82^ ^]^ Up to the present, such processes cannot be studied with time‐resolved X‐ray imaging in 3D due to the lack of temporal resolution. Salvo et al. measured the solidification of Al–Si–Fe–Cu at a cooling rate of 5 K s^−1^, however with a limited temporal resolution of 6.6 tps.^[^ ^45^ ^]^

**Figure 4:**
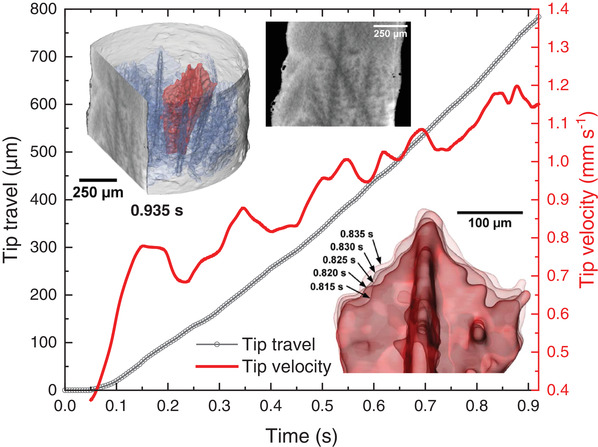
Evolution of aluminum dendrites in AlGe10 alloy during solidification at a cooling rate of 17 K s^−1^ and recorded continuously at 200 tps during a period of 60 s. Upper left inset: Rendered 3D image of a tomogram taken at *t* = 0.935 s showing Al‐rich dendrites in blue except for a selected one highlighted in red. The liquid phase is kept transparent. A virtual vertical cut is shown on the left of the rendered volume and separately provides a 2D view of the tomogram. Lower right inset: Magnification of the dendrite tip marked in red in the upper inset showing five consecutive, overlapping tomograms separated by a time increment of 5 ms. Graphs: Quantitative analyses of the tip travel distance smoothed over 100 ms (in black) and of the corresponding tip velocity (in red) over time (Video S2, Supporting Information).

In the example shown here, the morphological evolution of dendrites (shown in blue in **Figure** **4**) in the aluminum matrix (made transparent) is visualized (Video S2, Supporting Information). Moreover, the shape, tip travel distance, and tip velocity evolution of a selected dendrite (shown in red in Figure 4) averaged over 100 ms are determined quantitatively and also given in Figure 4. We observe that this particular dendrite tip grows by 775 µm in 0.83 s and its velocity accelerates from ≈800 to ≈1200 µm s^−1^ during that period. The maximum velocity of the dendrite corresponds to a motion of 6 µm per tomogram, which is in the same range as the spatial resolution. Thus, the high acquisition rate of 200 tps applied here is essential. Dendrite growth acceleration is due to increasing melt undercooling^[^ ^78^, ^79^, ^83^ ^]^ and the fluctuations are possibly the result of concentration or undercooling variations in front of the solid–liquid interface. The strength of tomoscopy in this example is obvious: It provides 3D information and trajectories in fast evolving samples that have not been accessible before.

### 3.3 Burning of a Sparkler Resolved at 400 tps

Exothermal combustion reactions, also called self‐propagating high‐temperature synthesis, are technologically important and accompanied by the release of a large amount of heat and a combustion wave front moving fast at typically 1–100 mm s^−1^.^[^ ^84^ ^]^ High temperatures and combustion rates prevent most real‐time, in situ investigations of phase transformations and combustion kinetics by conventional techniques. One of the crucial issues is to understand the role of liquid constituents in the combustion process.^[^ ^84^ ^]^ An example for this are pyrotechnic compositions like the ones used in sparklers, with burning velocities of more than 5 mm s^−1^ depending on the composition.^[^ ^85^, ^86^ ^]^ The number and type of ingredients used can vary a lot for differently colored sparks, different targeted burning rates, and also to influence the odor during burning.^[^ ^85^, ^87^ ^]^ One of the first patents in 1933 claims that sparklers are composed of dextrin (10%), Al (5%), Fe (30%), and Ba(NO_3_)_2_ (55%) as an oxidizer.^[^ ^87^ ^]^ The composition of modern sparklers is further described in the literature, but may differ depending on the suppliers.^[^ ^85^, ^86^ ^]^ The binders have a dual function in that they bind together the ingredients into a solid stick to facilitate handling, and further in that they act as fuels to promote combustion. The accelerators serve to increase the burning rate. During burning, iron particles cause the characteristic sparkling effect, hence the name of the product.^[^ ^88^ ^]^

**Figure 5:**
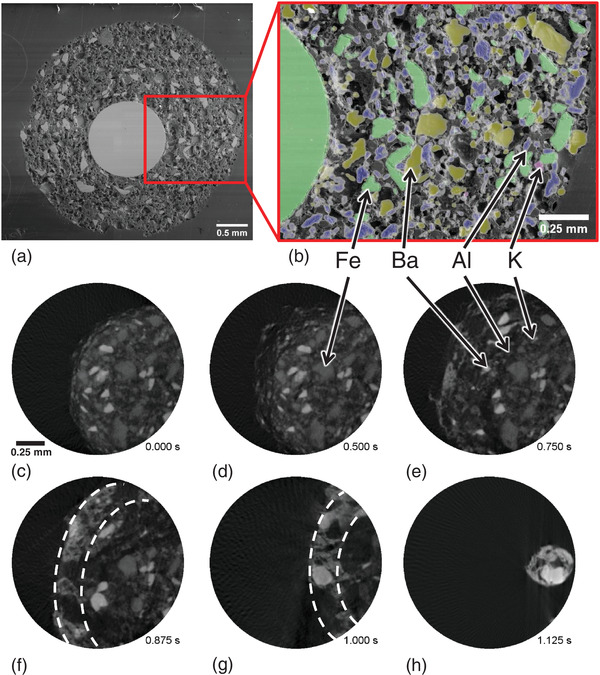
SEM images of a) the whole cross section of a sparkler and b) of a magnified segment overlapped with a colored EDX mapping (iron represented in green, aluminum in blue, barium in dark yellow and potassium in purple). c–h) X‐ray tomographic 2D slices representing different times (total acquisition period was 1.125 s) extracted from a tomoscopy sequence recorded at 400 tps after ignition (*t*_0_ = 0 s) of the sparkler. Broken white curves in (f) and (g) show the 25‐µm thick outer layer with increased BaO/BaO_2_ concentration (Video S3, Supporting Information).

SEM images and EDX mappings of the cross section of a sparkler (**Figure** **5**) show their composition: Iron (in green), aluminum (in blue), and barium nitrate (in yellow) as well as some potassium (in purple) in KNO_3_ or KClO_3_, which are conventionally used for sparklers.^[^ ^85^, ^89^ ^]^ The latter compound contains, together with the binder, the required oxygen for initiating the reaction of iron with the gaseous oxygen of the atmosphere.^[^ ^88^, ^89^, ^90^ ^]^

The tomoscopy experiment recorded with 400 tps shows for the first time the inside of a sparkler during burning in 3D. We identify the constituents and follow their decomposition, melting, and reactions (Figure 5c and Video S3, Supporting Information). The sparkler ignition starts at *t*_0_ = 0 s and is followed by a rapid volume growth by ≈56 % during the first 0.8 s caused by porosity formation in the binder as a consequence of heat and gas generation. In parallel, the porous binder and aluminum powder particles start burning due to the presence of the oxidizing agent KClO_3_ in addition to oxygen from the surrounding atmosphere.^[^ ^89^ ^]^ Aluminum powders can burn above ≈576 °C depending on their particle sizes.^[^ ^91^ ^]^ Ba(NO_3_)_2_ decomposes to BaO (*T*_m_ = 1918 °C) above 550 °C, reacting with the available oxygen to barium peroxide between 500 and 600 °C,^[^ ^89^, ^90^ ^]^ which melts at 450 °C. Judging the “acrid odor,”^[^ ^85^ ^]^ formation of NO*_x_* is likely. As the reaction can occur without sparks in other atmospheres^[^ ^90^ ^]^ one assumes that barium nitrate and the metals react to BaO, NO_2_, and metal oxide.^[^ ^90^ ^]^

At ≈500 °C iron powders start burning as their ignition temperature is found to range from 428 to 555 °C.^[^ ^92^ ^]^ The burning iron particles lose their bond to the binder and are rapidly ejected from the sparkle, thus giving rise to the characteristic sparkling effect. The radially flying iron particles suddenly disappear from the tomograms and cannot be imaged as they no longer follow the required rotation of the stage. The strong exothermic reaction promotes rapid combustion, increases the temperature, and leads to a volume reduction of more than 90% relative to the initial volume in the following 0.5 s. During that period an accumulation of BaO/BaO_2_ in the porous sparkler structure is observed as a light grey layer of ≈25 µm thickness at the sparkler's surface, best observed in Figure 5c between 0.8 and 1 s. Eventually, the burning terminated at *t* = 1.125 s, but at still high enough temperature a trapped, molten barium nitrate droplet can be observed swashing in the carbonized sparkler skeleton (Figure 5c and Video S3, Supporting Information). A more detailed overview of the possible reactions is contained in the Supporting Information.

### 3.4 Foaming of AlSi6Cu4 Alloy Resolved at 650 tps

Metallic foams are promising materials for a range of applications due to a combination of properties such as low weight, high specific strength and stiffness, and energy absorption capability.^[^ ^93^ ^]^ Driven by demands of industry, great efforts have been undertaken to improve foam quality. However, large‐scale production has been hindered by an incomplete understanding of the processes by which foaming metals are stabilized, how liquid foams solidify, and how these processes influence the final structure of the foam. To reach a uniform structure is still a major challenge. During metal foam evolution, liquid metal flows and films are created, which in the course of foam expansion become thinner. A lack of film stability eventually may lead to film rupture and corresponding bubble coalescence.^[^ ^94^ ^]^ The result is a non‐uniform pore size distribution in the solid metal foam. To examine the molten system in situ and to verify and understand proposed mechanisms sophisticated experimental setups are required.^[^ ^22^ ^]^

**Figure 6:**
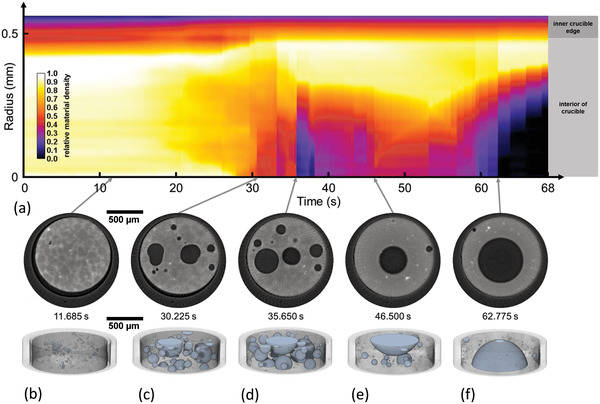
Evolution of a liquid AlSi6Cu4 alloy foam recorded continuously at 650 tps over a period of 68 s. a) Relative material density calculated from measured X‐ray intensities averaged over the sample height as a function of radial position showing dense regions in light colors and bubbles in black. b–f) 2D slices extracted from center of the corresponding 3D tomograms selected at five different times. b) First bubbles grow after nucleation. Al dendrites in the precursor are still visible. c,d) Bubble evolution and coalescence in a still homogeneous gas and material distribution, the latter partially molten. e,f) Influence of radial *g*‐forces on the weakly stabilized now fully liquid foam leads to a mass and gas re‐allocation and eventually the formation of a single large, centered gas bubble (except for a small satellite bubble pinned to the oxide layer of the sample surface) (Video S4, Supporting Information).

To analyze the foaming process of a thixocast AlSi6Cu4 + 0.8 wt% TiH_2_, alloy we heat up a sample to 625 °C at a rate of 2.15 K s^−1^. We start the tomoscopy measurement after reaching 485 °C, define this as *t*_0_ = 0 s and observe the sample evolution in terms of material diffusion, gas nucleation, bubble growth, coarsening, and coalescence over more than 1 min (**Figure** **6** and Video S4, Supporting Information), thus demonstrating the capability of the method at such high speeds over a long period of time.

Full 3D images and representative 2D slices of the sample in the stage of transition to the liquid‐dominated foam show predominantly round pores throughout the experiment, see Figure 6b–f. After 11.685 s the TiH_2_ particles (light spots), the nucleation of first bubbles (dark spots) and a Cu‐rich mesh decorating the grains (light lines) are observed in the selected slice extracted from the tomoscopy experiment as shown in Figure 6b. After 30.225 s, at 553 °C, randomly distributed bubbles evolve and the higher copper content at the grain boundaries starts to vanish due to element interdiffusion and corresponding alloy formation (Figure 6c). Copper diffusion continues until ≈50 s, at 595 °C, where a final homogeneous alloy is formed and the copper decoration has completely vanished (Figure 6d,e). At this stage, bubble nucleation occurs not directly at the TiH_2_ blowing agent particles but at the grain surfaces which contain more copper, leading to a lower liquidus point than the rest of the matrix, a mode called type‐II in the literature.^[^ ^95^ ^]^ But in contrast to powder metallurgical precursors obtained by the compaction of elemental powders, no elemental silicon particles are present in thixocast precursors and therefore the regions of lower liquidus temperature are not found close to silicon particles as reported for powder metallurgical precursors.^[^ ^95^ ^]^ Liquid foams obtained from thixocast precursors are known to have rounder but less stable bubbles,^[^ ^96^ ^]^ and this is obvious from the images. This material was deliberately selected to demonstrate how *g*‐forces generated by the sample rotation might influence the morphological evolution of the sample. To show this in a more quantitative way, Figure 6a specifies the height‐averaged X‐ray intensities of the sample during foaming for different distances from the center of rotation. At 650 tps, the crucible rotates at 325 Hz, which corresponds to a centrifugal force of 212 × *g* at 0.5 mm radius. The relative material density distribution is still uniform within the first 30 s as long as the sample is still mostly solid, indicated by non‐spherical bubble shapes. Thus, rotational forces hardly influence foam morphology at that point. Between 35 and 38 s, some bubble agglomeration over the whole sample is observed reflected by a low relative density close to the rotation center (Figure 6a). The bubbles seem to redistribute radially again until 45–50 s, indicating a transient stage of a bubble created near the center. At this temperature, 584–594 °C, a still notable solid fraction of ≈35% is expected,^[^ ^53^ ^]^ which allows for the outward bubble movement in the course of bubble growth. Foam ageing and bubble coalescence set in and the relative material density is finally clearly influenced by the rotational forces, which lead to the formation of a single large central bubble for >60 s as shown in Figure 6a,f. A few small satellite bubbles at the sample edge are also observed. They are most likely pinned to oxide particles that allow them to maintain their position despite gravity levels above 200 × *g*. At this point, foam evolution is dominated by centrifugal forces and the results have to be interpreted carefully. As mentioned above, this effect is especially strong in this foam type chosen with an intention, while other foam types are not so prone to such effects as shown in ref. [22] and also in the next example. Note that the rapid rearrangements of bubbles especially in the stages between Figure 1b,c can be resolved only because the tomoscopy acquisition rate is so high.

### 3.5 Coalescence of Bubbles in AlSi8Mg4 Alloy Resolved at 1000 tps

Coalescence of two bubbles in a liquid metal foam is an undesired but unfortunately very common phenomenon leading to an ageing of its cellular structure. It is induced by the rupture of the liquid film separating two bubbles, which takes place due to a lack of stability. The stability of liquid films in foams produced by the powder metallurgical route is provided by oxide networks resident in the metal powders involved.^[^ ^97^ ^]^ Previous radioscopy and tomoscopy experiments revealed a film rupture time of <1 ms and a coalescence time (the time to form a new bubble without a neck or straight sections) of 0.5–1.2 ms.^[^ ^22^, ^98^ ^]^ Such time scales are now accessible with the temporal resolution of the current tomoscopy technique.

**Figure 7:**
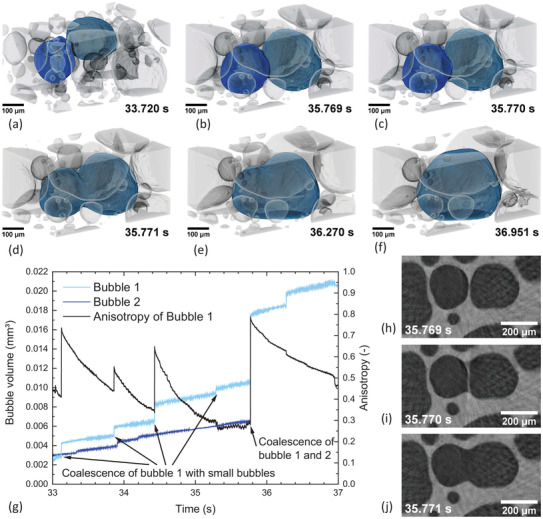
3D rendering of the gas bubble arrangement in a liquid AlSi8Mg4 foam recorded with 1000 tps. The metallic matrix is kept transparent and the bubbles are colored (inverted contrast). a) Two separated bubbles marked in cyan and blue. b–d) 3D sequence of the rupture of the separating film in just <1 ms followed by the coalescence of the two bubbles to one. e,f) Relaxation of the resulting bubble by adopting a more spherical shape in the next ≈1.2 s. g) Bubble volumes and anisotropy of bubble 1 over time (Video S5, Supporting Information). h–j) Horizontal slices showing the sequence of the rupture of the separating film.

We therefore apply tomoscopy at a rate of 1000 tps to visualize the approach and merger of two bubbles in the early foaming stage of an AlMg8Si4 foam (**Figure** **7** and Video S5, Supporting Information), featuring various stages. First we notice that two bubbles initially (at 33.720 s) separated by a gap of ≈50 µm get in touch with each other within a period of ≈2 s (at 35.769 s, Figure 7b). The quantitative analysis of bubble volumes shown in Figure 7g gives a hint what the driving force of this approach might be: As especially the cyan‐colored (right) bubble in Figure 7a more than doubles its volume from 0.0049 to 0.0105 mm^3^ in this short period, it is likely that coalescence with several small bubbles, seen by the small jumps in bubble volume in Figure 7g, and local gas generation have inflated the bubble and have shifted it toward the other one that also has increased in volume, albeit to a lesser extent. These small coalescence events temporarily increase the anisotropy of the absorbing bubble, grey line in Figure 7g. After the bubbles touch each other (Figure 7b), the actual film rupture (at 35.770 s, Figure 7c) is very fast in accordance with previous reports^[^ ^22^, ^98^ ^]^ and a neck is formed. The anisotropy more than doubles instantaneously as shown in Figure 7g. Just 1 ms later at 35.771 s (Figure 7d) we see a widened neck, indicating a hole in the film of more than 100 µm diameter. After further 0.5 s, the neck between the bubbles has evolved into a straight line (at 36.270 s, Figure 7e) and an elongated bubble with an anisotropy of 0.63 has emerged, which then is converted into a more spherical bubble with an anisotropy of ≈0.5 after further 0.7 s (at 36.951 s, Figure 7f). The relaxation times of anisotropy are longer than reported in previous work based on radioscopy,^[^ ^22^, ^98^ ^]^ but the scenarios are very different here as bubbles are small in the very early stage investigated and the melt is more viscous due to a lower foaming temperature of ≈550 °C, which still allows for some solid fraction in the foam.

We also observe in this experiment (Figure 7) a much smaller influence of centrifugal forces than in the previous example (Figure 6). This is due to the different alloy and the applied powder metallurgical manufacturing route of the precursor that involves compressing powder particles while maintaining their oxidized surfaces. This causes a gel‐like and less deformable consistency of the liquid and a higher resistance to centrifugal forces.

### 3.6 Further Increase of Acquisition Rate and Alternative Directions

To visualize the actual rupture process and, for example, to measure the velocity of the retracting liquid film in 3D, even higher acquisition rates (about ten times) would be necessary based on the experience with radioscopy experiments.^[^ ^98^ ^]^ From the perspective of X‐ray imaging this could be possible, but the resulting 100 times higher *g*‐forces would most likely dominate and strongly compress the foam. This implies that for this application we have reached a physical limit. For other applications, however, a further (brute force) increase of acquisition rate by faster rotation might be reasonable.

A possible solution for the centrifugal forces problem could be a multibeam approach with stationary samples. Some approaches have recently been discussed.^[^ ^99^, ^100^ ^]^ Another possibility is applying a reduced number of projections or limited angle acquisition, and combining with interlaced or intelligent reconstruction algorithms.^[^ ^101^, ^102^, ^103^ ^]^

In addition to the study of reactions and transformations controlled by temperature, tomoscopy could also be applied to processes controlled by electrical currents, for example, charge, discharge, or failure of batteries, or to mechanical phenomena such as dynamic deformation or impact of materials.

## 4 Conclusions

A new tomoscopy setup is presented that is capable of recording up to 1000 tomograms in a second (1000 tps) with a voxel size of 2.75 µm and a measured spatial resolution of 7.6–8.2 µm depending on the rotation velocity and distance to the center. This rate can be sustained for minutes. With this setup new quantitative insights into fast phenomena in materials are possible as demonstrated in various cases:

• Bi‐rich droplets in a solidifying monotectic alloy are observed as they form in an undercooled Al‐Bi10 alloy, move, grow, and undergo Ostwald ripening. 3D imaging allows us to locate them as they get trapped between Al‐rich dendrites.
• Dendrite growth in a solidifying Al‐Ge10 model alloy is studied in great detail during cooling at industrial rates. Their size, morphological changes, and growth direction are obtained for individual dendrites and their properties evaluated quantitatively at 5 ms temporal resolution.
• Self‐propagating high‐temperature reaction synthesis is followed in a commercial sparkler. Details of the reaction including the formation of a liquid phase are explored in situ.
• Metal foam created by decomposition of blowing agent is investigated by tomoscopy. In a first system, an AlSi6Cu4 alloy, Cu‐rich liquid is identified as the location at which bubbles form. This system represents a case where centrifugal forces influence an object at a certain evolution stage, unlike the other, a more stable AlSi8Mg4 alloy, which remains in shape despite the even higher forces. Tomoscopy at the highest rate of 1000 tps allows us to follow details of bubble coalescence and bubble shape relaxation in situ with millisecond temporal resolution.

These applications show that the influence of high radial *g*‐forces is a priori not negligible at such high rotation speeds and results might be affected. However, we have found cases where these forces do not prevent the use of tomoscopy.

## Conflict of Interest

The authors declare no conflict of interest.

## Supporting information

Supporting Information

Supplemental Video 1

Supplemental Video 2

Supplemental Video 3

Supplemental Video 4

Supplemental Video 5

## Data Availability

All original projections and selected reconstructed tomograms are available at the PSI Open Data Provider, https://doi.org/10.16907/d7582cb6-7850-42bc-ad76-e845b998e9ca. Further data can be made available upon reasonable request.
