# Supplementary material for: Tomoscopy: Time‐Resolved Tomography for Dynamic Processes in Materials
Source: Adv Mater. 2021 Sep 23;33(45):2104659. doi: 10.1002/adma.202104659 (PMC11468671; doi:10.1002/adma.202104659)
Supplement: Supplementary file 1 — Supporting Information [file ADMA-33-2104659-s001.pdf]

© 2021 Wiley-VCH GmbH

ADVANCED MATERIALS

Supporting Information

for *Adv. Mater.,* DOI: 10.1002/adma.202104659

Tomoscopy: Time-Resolved Tomography for Dynamic Processes in Materials

Francisco García-Moreno, Paul Hans Kamm, Tillmann Robert Neu, Felix Bülk, Mike Andreas Noack, Mareike Wegener, Nadine von der Eltz, Christian Matthias Schlepütz, Marco Stampanoni, and John Banhart*

Supporting Information

Spatial resolution

In order to determine the real imaging resolution of the tomoscopy setup (and not just the voxel size of the images) a test object (“Siemens star” calibration pattern on Si crystal) with known dimensions and high imaging contrast was chosen as proposed by Weiß et al.[104] and shown in **Figure S1a**. It has an outer diameter of 120 µm as marked by a green circle and it was placed 230 µm away from the centre of rotation. A tomographic slice recorded under optimized conditions at an acquisition speed of 1000 tps, with 5 µs exposure time per projection and 100 projections per tomogram, is shown in Figure S1b. A magnification of it with a red arrow indicating how the resolution line is drawn is shown in Figure S1c. Figure S1d shows the "unrolled" polar coordinate transformation (nearest-neighbour interpolation), in which the red line delimits the radius at which all the branches of the star are still visible. The intention is to obtain a value at which all lines can be distinguished from each other with a Michelson contrast of more than 5 %, although some of the lines already show higher contrast values even at lower line widths. This analysis yields the value given in the main paper, namely 8.2 µm for 1000 tps compared to 6.9 µm given by a previous analysis.[99] In general the influence of parameters such as image acquisition time, speed of rotation, material absorption or reconstruction parameters must also be taken into account. Furthermore, a contrast unevenly distributed over the pattern can be seen, which might be due to slight deviations from the angular accuracy of the rotation axis at these fast rotation speeds. As a result, determining an exact and generally valid value for the resolution is difficult, but the values derived here provide a good assessment.

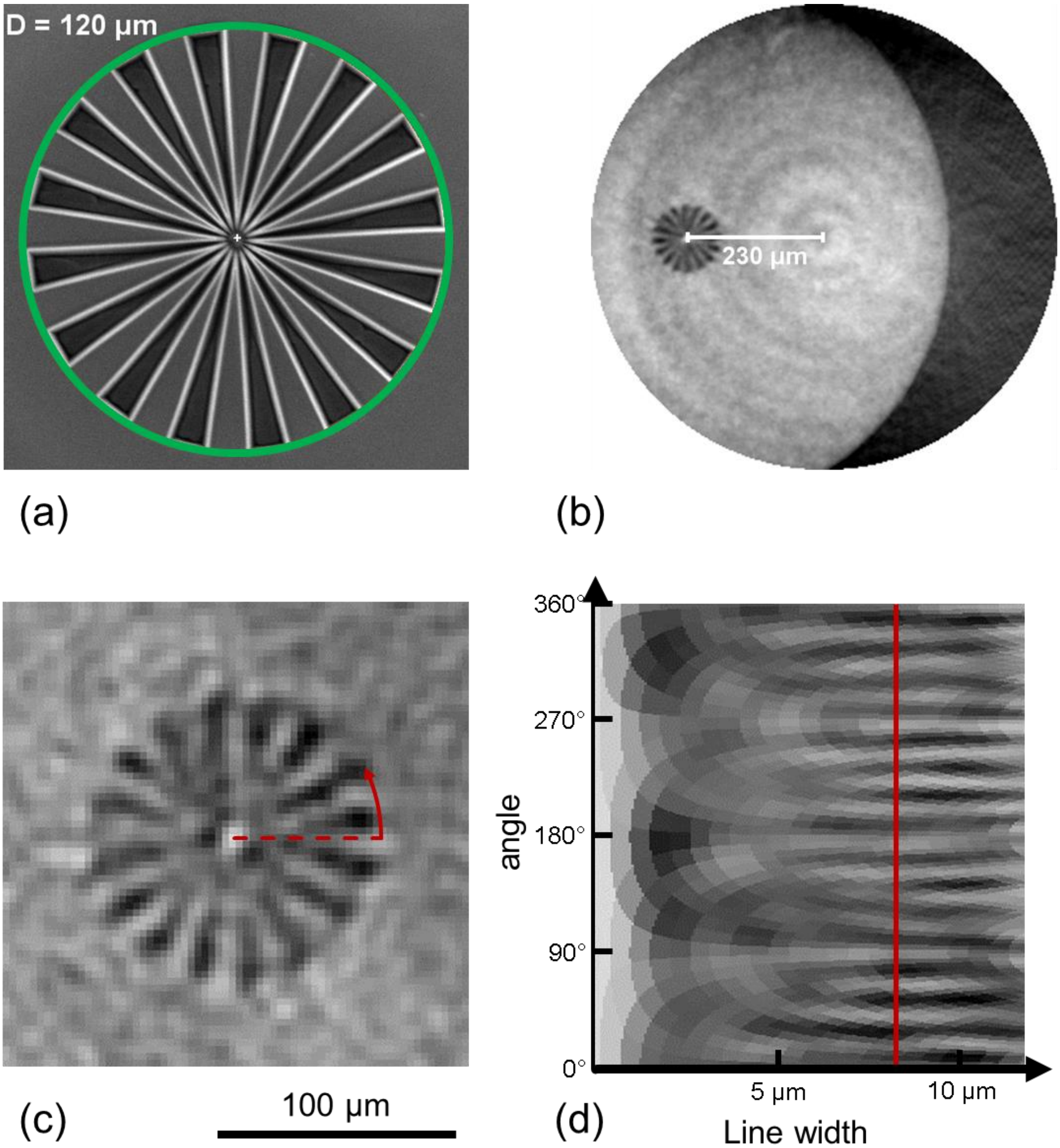

Figure S1. "Siemens star" calibration pattern on Si crystal with a diameter of 120 µm and an outermost line width of 11.8 µm. (a) SEM image and (b) tomographic slice showing the pattern position 230 µm away from the rotation centre. (c) Magnification of a tomographic slice recorded at 1000 tps acquisition speed, with 5 µs exposure time per projection and 100 projections per tomogram. (d) The "unrolled" polar coordinate transformation (nearest-

neighbour interpolation) shows the resolvable structure versus the angle with a contrast cut-off at an approximate line width of 8.2 µm for 1000 tps.

Radial acceleration

Large *g*-forces at high rotation speeds might occur depending on the rotation frequency and sample radius to the rotation axis according to $a_z = 4\pi^2 f^2 r$ as illustrated in Figure S2. A basic requirement of a tomoscopy study is that such *g*-forces should not affect the results beyond an acceptable threshold. Undesired effects could appear especially at large distances from the rotation centre and a possible influence of the rotation should be considered, especially for liquid samples. An example is the evolution of a weakly stabilised AlSi6Cu4 foam in Figure 6, where the *g*-forces are not relevant during gas nucleation, but gain importance later during foam evolution.

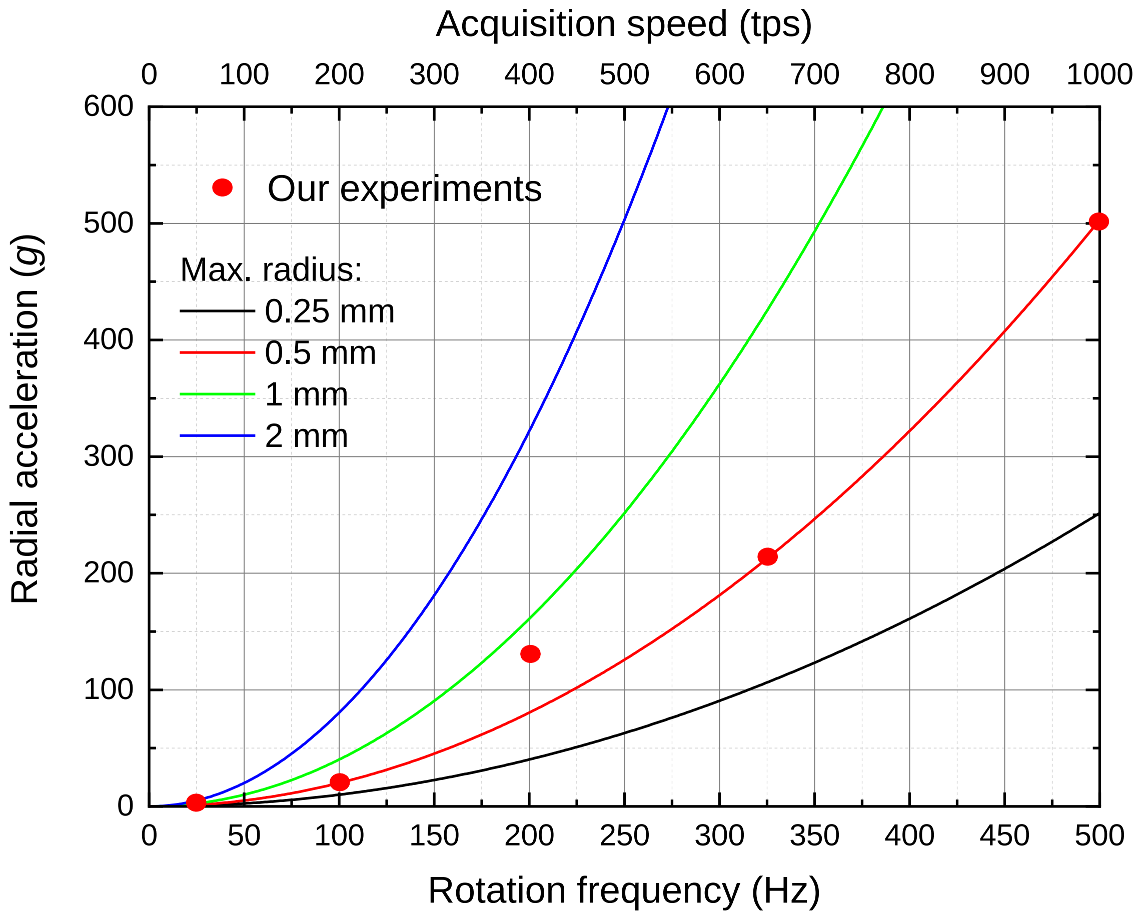

Figure S2. Radial acceleration acting on the rotating samples during tomoscopy as a function of the acquisition speed and rotation frequency for different radial positions. The parameter of experiments presented in the present study (at different rotation frequencies) are marked with red dots.

Reactions in a burning sparkler

The following possible reactions to form sparks are:[82, 83]

$$2\mathrm{Ba(NO_3)_2}\ (\mathrm{s}) \rightarrow 2\mathrm{BaO}\ (\mathrm{s}) + 2\mathrm{N_2}\ (\mathrm{g}) + 5\mathrm{O_2}\ (\mathrm{g})$$

$$2\mathrm{KClO_3}\ (\mathrm{s}) \rightarrow 2\mathrm{KCl}\ (\mathrm{s}) + \ 3\mathrm{O_2}\ (\mathrm{g})$$

$$4\mathrm{Al}\ (\mathrm{s}) + \ 3\mathrm{O_2}\ (\mathrm{g}) \rightarrow 2\mathrm{Al_2O_3}\ (\mathrm{s})$$

$$4\mathrm{Fe}\ (\mathrm{s}) + \ 3\mathrm{O_2}\ (\mathrm{g}) \rightarrow 2\mathrm{Fe_2O_3}\ (\mathrm{s})$$

$$3\mathrm{Fe}\ (\mathrm{s}) + \ 2\mathrm{O_2}\ (\mathrm{g}) \rightarrow \mathrm{Fe_3O_4}\ (\mathrm{s})$$

The reactions between barium nitrate and the metal powders are:[83]

$$10\mathrm{Al}\ (\mathrm{s}) + \ 3\mathrm{Ba(NO_3)_2}\ (\mathrm{s}) \rightarrow 3\mathrm{BaO}\ (\mathrm{s}) + 3\mathrm{N_2}\ (\mathrm{g}) + 5\mathrm{Al_2O_3}\ (\mathrm{s}),$$

$$15\mathrm{Fe}\ (\mathrm{s}) + 4\mathrm{Ba(NO_3)_2}\ (\mathrm{s}) \rightarrow 4\mathrm{BaO}\ (\mathrm{s}) + 4\mathrm{N_2}\ (\mathrm{g}) + 5\mathrm{Fe_3O_4}\ (\mathrm{s})$$

One can assume by the “acrid odour”[78] that NO_x is formed. As the reaction can occur without sparks in other atmospheres[83] one can suppose that barium nitrate and the metals react as following (simplified with the assumption that all NO_x will be NO_2):[83]

$$2\mathrm{Al}\ (\mathrm{s}) + \ 3\mathrm{Ba(NO_3)_2}\ (\mathrm{s}) \rightarrow 3\mathrm{BaO}\ (\mathrm{s}) + 6\mathrm{NO_2}\ (\mathrm{g}) + \mathrm{Al_2O_3}\ (\mathrm{s}),$$

$$3\mathrm{Fe}\ (\mathrm{s}) + \ 4\mathrm{Ba(NO_3)_2}\ (\mathrm{s}) \rightarrow 4\mathrm{BaO}\ (\mathrm{s}) + 8\mathrm{NO_2}\ (\mathrm{g}) + \mathrm{Fe_3O_4}\ (\mathrm{s}).$$

The required oxygen is taken from the oxidizers (here potassium chlorate) or from the surrounding atmosphere.[82, 83] BaO (T_m = 1918 °C) reacts to Bariumperoxide between 500 °C and 600 °C.

Al-Bi phase diagram

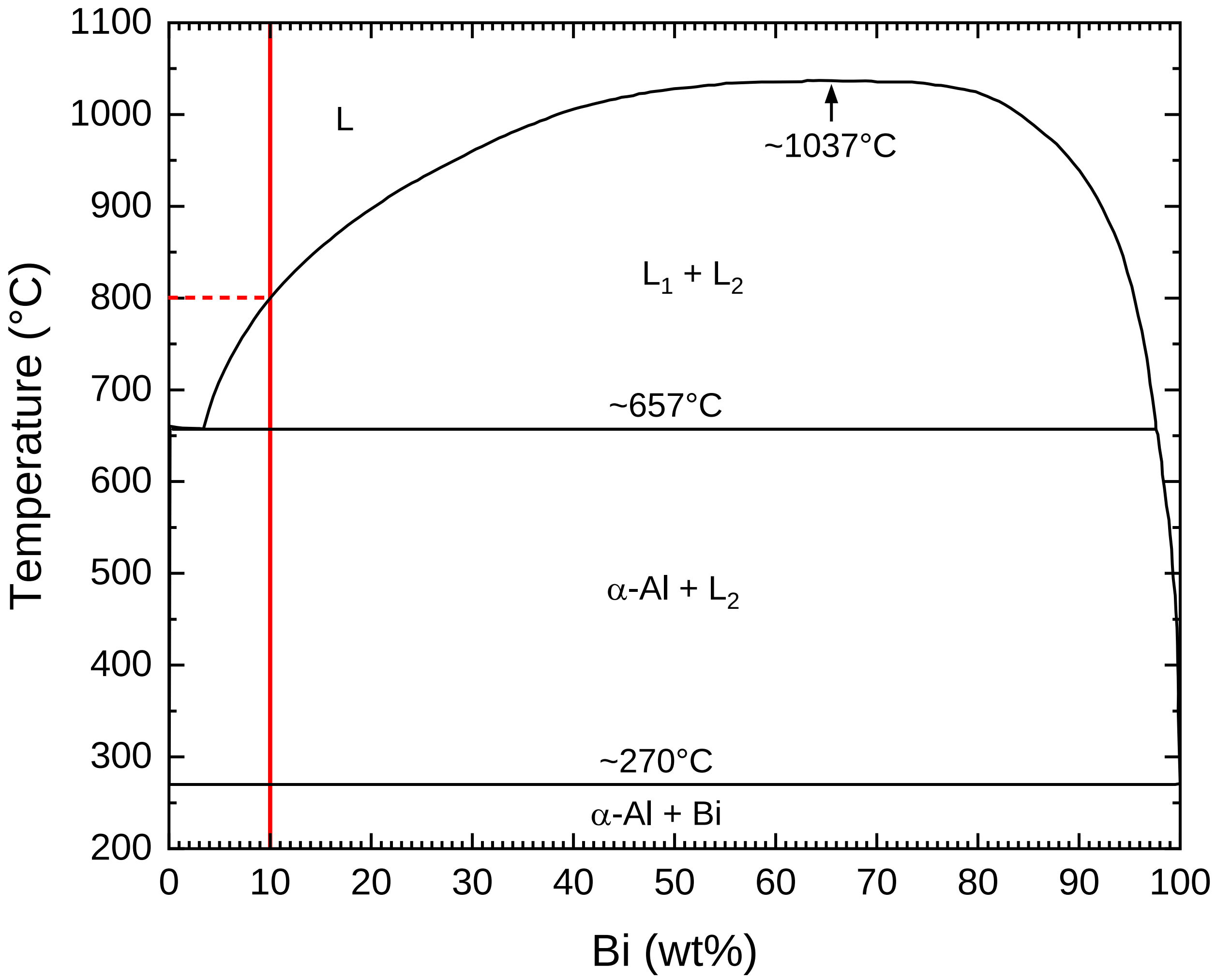

Figure S3. Al-Bi phase diagram adapted from Massalski.[52] Our composition (AlBi10) is marked by a red line.

Temporal evolution of mean diameter of Bi-rich droplets

Visual inspection of Figure 1(d)–(h) already suggests coarsening of the Bi-rich droplets formed during cooling of the alloy. In order to further elucidate this we calculated the equivalent diameters of all the droplets in each tomogram and then determined a mean diameter. We used a larger box than the one shown in Figure 1 to include more particles and to improve statistics. Figure S4 displays the mean Bi droplet diameter as a function of time

together with the sample temperature. With the exception of the first 2 s that are dominated by a few large droplets at the top of the melt, the mean diameter increases continuously from 4.9 µm to 6 µm, which corresponds to almost a doubling of the mean volume. At first sight, this behaviour resembles Ostwald ripening, but an attempt to apply LSW theory does not yield a good approximation. Moreover, the particle size distributions (not shown) do not follow the typical trend given by LSW theory other than, for example, in some oil/water emulsions where this is the case.[105] There is a tail of too large particles from the beginning and many very small particles can be only insufficiently resolved properly as they are too close to the resolution limit of tomoscopy. The non-constant temperature and the wealth of forces acting on the droplets including hydrodynamic forces as discussed in the main paper also have to be considered. The simple conditions for the validity of LSW theory are therefore not fulfilled in this experiment.

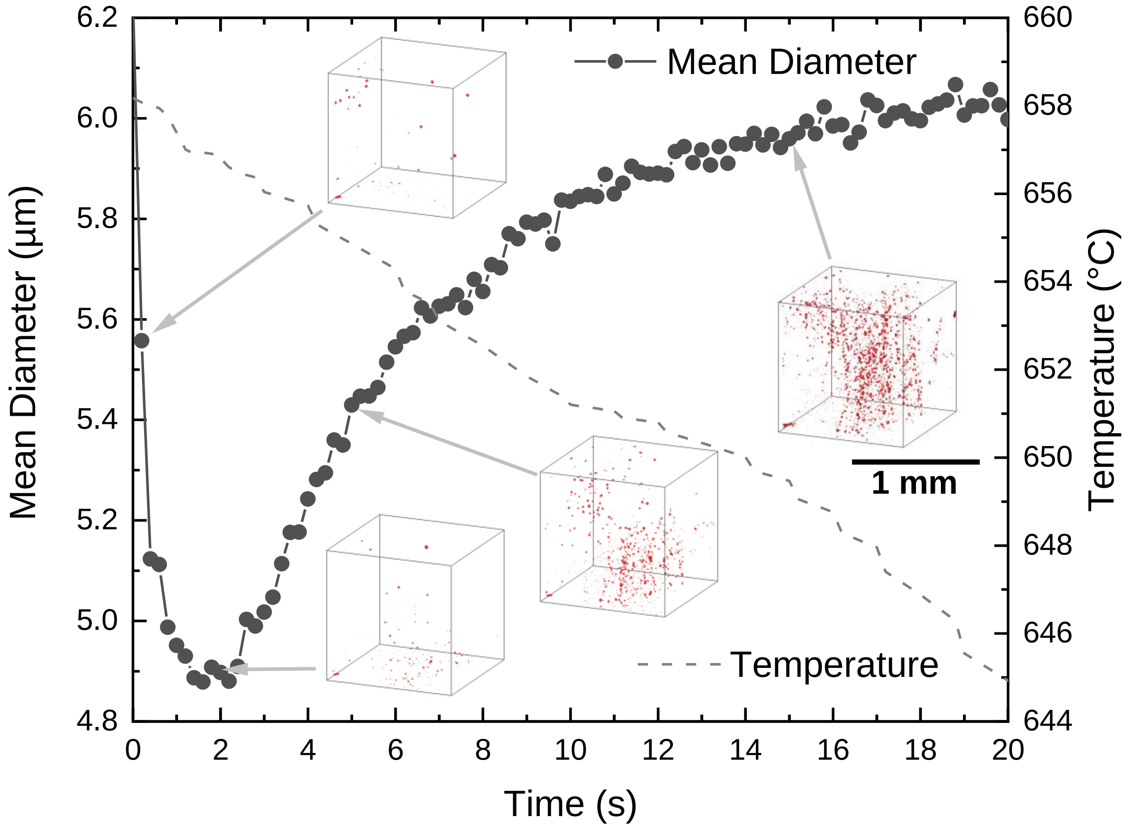

Figure S4. Temporal evolution of the mean diameter of Bi-rich droplets in a selected volume in the centre of the AlBi10 sample shown in Figure 1 and the corresponding sample temperature. The insets show tomograms of the Bi-rich droplets (in red) at selected times.
